# Emerging Copper-to-Copper Bonding Techniques: Enabling High-Density Interconnects for Heterogeneous Integration

**DOI:** 10.3390/nano15100729

**Published:** 2025-05-12

**Authors:** Wenhan Bao, Jieqiong Zhang, Hei Wong, Jun Liu, Weidong Li

**Affiliations:** 1 Department of Electrical Engineering, City University of Hong Kong, Kowloon, Hong Kong SAR, China; wenhanbao2-c@my.cityu.edu.hk; 2 Hubei Jiu Feng Shan Laboratory, Wuhan 430074, China; liujun@jfslab.com.cn; 3 Yangtze Memory Technologies Co., Ltd., East Lake High-Tech Development Zone, Wuhan 430078, China; weidong_li@ymtc.com

**Keywords:** Moore’s Law, heterogeneous integration, Cu-Cu bonding, high density interconnects, thermal budget

## Abstract

As CMOS technology continues to downsize to the nanometer range, the exponential growth predicted by Moore’s Law has been significantly decelerated. Doubling chip density in the two-dimensional domain will no longer be feasible without further device downsizing. Meanwhile, emerging new device technologies, which may be incompatible with the mainstream CMOS technology, offer potential performance enhancements for system integration and could be options for a More-than-Moore system. Additionally, the explosive growth of artificial intelligence (AI) demands ever-high computing power and energy-efficient computing platforms. Heterogeneous multi-chip integration, which combines diverse components or a larger number of functional blocks with different process technologies and materials into compact 3D systems, has emerged as a critical pathway to overcome the performance limitations of monolithic integrated circuits (ICs), such as limited process/material options, low yield, and multifunctional design complexity. Furthermore, it sustains Moore’s Law progression for a further smaller footprint and higher integration density, and it has become pivotal for “More-than-Moore” strategies in the next CMOS technology revolution. This approach is also crucial for sustaining computational advancements with low-power dissipation and low-latency interconnects in the coming decades. The key techniques for heterogeneous wafer-to-wafer bonding involve both copper-to-copper (Cu-Cu) and dielectric-to-dielectric bonding. This review provides a comprehensive comparison of recent advancements in Cu-Cu bonding techniques. Major issues, such as plasma treatment to activate bonding surfaces, passivation to suppress oxidation, Cu geometry, and microstructure optimization to enhance interface diffusion and regrowth, and the use of polymers as dielectrics to mitigate contamination and wafer warpage, as well as pitch size scaling, are discussed in detail.

## 1. Introduction

(a) Heterogeneous Integration as an Option for More Moore and More Than Moore

There is a general consensus that the next “more Moore” advancement should focus on 3D stacking and the heterogeneous integration of multiple “chiplets” within a single package [1,2,3,4]. The concept of vertical stacking dates back to the 1960s with the introduction of flip-chip technology, initially used to connect multi-chip modules in mainframe computers [5]. Today, this technology is widely applied across various levels of the electronic system hierarchy, from the package level to the system level, as exemplified by the system-on-chip (SoC) devices used in mobile phones [1,2,3,4].

At the device level, flash memory technology has made significant progress. Three-dimensional stacking has enabled tera-scale memory, with the latest 3D NAND flash reaching over 230 layers [4]. However, adding more layers seems to have a diminishing effect on chip density. For logic circuits, the Complementary Field-Effect Transistor (CFET), which stacks an nMOS on top of a pMOS, has been proposed as an emerging option to continue logic scaling into the next decade. This technique can save up to 50% of the footprint of a CMOS cell while improving the ON–OFF current ratio [4].

At the system level, flip-chip technology involves depositing metal bumps on IC bond pads at the wafer level during back-end-of-line (BEOL) processing. Unlike wire bonding, where chips are connected using fine wires, flip-chip technology flips the chips over a substrate, aligning the bumps on the chips to corresponding bond pads on the substrate. This method is commonly used in high-performance graphics cards and is often referred to as 2.5D technology [5].

**Figure 1:**
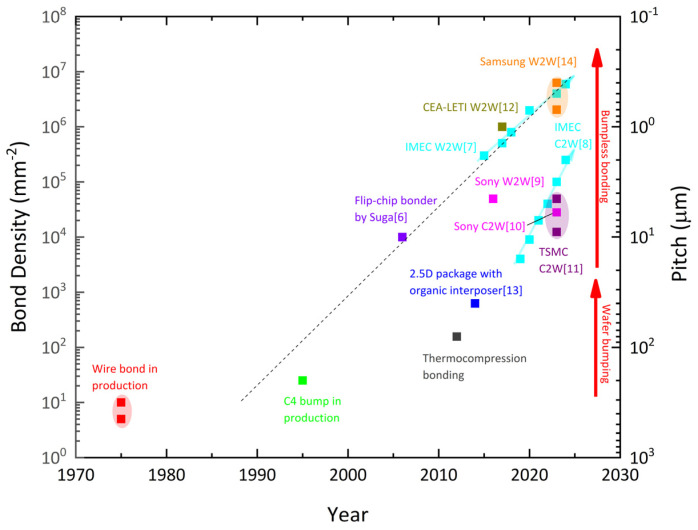
Technology trend on the pitch scaling and bond density enhancement over the last five decades.

The evolution of this technology has led to the development of chip-level or monolithic 3D heterogeneous integration technology. Figure 1 showcases key milestones in high-density interconnect technology innovation. Beginning with early wire bonding in the 1970s, which featured a relatively low bonding density of roughly a dozen bonds per square millimeter, advancements in interconnect technology have significantly increased density over time. The introduction of flip-chip technology led to an order-of-magnitude improvement in bonding density [6]. More recently, sophisticated hybrid bonding techniques, including wafer-to-wafer (W2W) and chip-to-wafer (C2W) hybrid bonding [7,8,9,10,11,12,13,14], have emerged to meet the growing demand for compact device architectures, high-speed inter-module communication, and enhanced interconnect density as device scaling continues. For instance, marking an industry milestone in 2016, Sony successfully implemented hybrid bonding for mass-produced image sensors, establishing chip-stacking architecture with pixel arrays vertically interconnected to logic circuits through micrometer-pitched copper interconnects [9,10]. The technology was subsequently scaled by leading semiconductor manufacturers such as TSMC and Samsung for 3D IC packaging applications, demonstrating breakthrough capabilities in interconnect miniaturization (pitch size <10 μm) and high-density integration (~10,000 interconnects/mm^2^) through optimized hybrid bonding processes [11,14]. Over the past two years, continuous refinements in hybrid bonding have pushed beyond conventional scaling limits and enabled unprecedented 3D integration densities. Cutting-edge research by IMEC realized 400 nm pitch direct interconnect bonding at the wafer scale in 2024, achieved through iterative process optimizations in overlay alignment and surface planarization [7]. The pitch size reduction process of hybrid bonding starts from 10 μm of aluminum interconnect, passes through micrometer breakthrough of copper interconnect, and, finally, moves forward to the nanometer level. Its development is not only dependent on material and process innovation (such as CMP, plasma cutting) but is also directly driven by the needs of high-performance computing, such as AI and high bandwidth memories. In the future, this technology will play an essential role in 3D packaging and system-level integration. With the accumulation of technology over the last five decades, 3D stacking technology has become quite mature. Instead of stacking different dies to create 3D ICs, monolithic 3D ICs start with a base wafer, onto which additional layers of semiconductor materials, metal layers, and active and passive circuitry are integrated using conventional IC fabrication processes. This approach allows for more efficient and compact designs, enhancing performance, functionality, and economics. Heterogeneous integration (HI) has been recognized as the key option for “more Moore” and post-Moore semiconductor technology [15,16].

Three-dimensional heterogeneous integration technology also facilitates the integration of non-digital circuits for system integration. On top of silicon CMOS technology, mixed-signal integrated circuits (ICs) incorporate sensors and analog/RF functionalities through silicon vias (TSVs) [3,16,17,18]. Jeong et al. detailed the implementation of heterogeneous integration of RF InGaAs high-electron-mobility transistors (HEMTs) on a CMOS substrate [17,18]. III–V compound semiconductors are renowned for their high-frequency application potential, while silicon CMOS excels in high integrated density. The synergistic monolithic integration of III–V and Si electronics offers significant advantages, prompting ongoing efforts to achieve this integration. Accordingly, Jeong et al. [17] achieved a high unity current gain cutoff frequency of 448 GHz and a unity power gain cutoff frequency of 742 GHz with their reported technology. Thus, heterogeneous integration would result in a new paradigm through the vertical integration of multi-material systems (Si/III-V/2D materials), multi-process node components (e.g., 5 nm CMOS + 28 nm analog), and multifunctional modules (logic, memory, RF, photonics) via advanced system-in-package (SiP) techniques, such as silicon interposers, wafer bonding, metal interconnection, and micro-bump arrays [7,8,9,10,11,12,13,14,15]. This novel technology overcomes the limitations of conventional system-on-chip (SoC) architectures by achieving functional heterogeneity, bandwidth enhancement, energy efficiency, scalability, and reconfigurability. TSMC’s CoWoS and Intel’s Foveros are good examples. In particular, the recent Foveros-based Intel CPU, Lakefield, used a novel method for connecting various chips on a substrate [19]. This technology enables the interconnection of CPU and memory chips through silicon vias, bridges, silicon interposers, or wiring layers, and then packages them into a single chip. This technology has been considered the primary technology for achieving trillion-level integration in the coming decades due to its flexibility, scalability, and integration efficiency. The “near-memory computing” architecture, in particular, boosts data processing speed while reducing power dissipation, which is crucial for AI platforms.

The latest report on the NVIDIA Grace Hopper graphic accelerator highlights its extremely high performance and energy efficiency [20] while catalyzing emerging fields, like high-density and high-speed silicon photonics integrated circuits with 1.6 Tbps optical I/O and quantum computing with cryogenic 3D interconnects. By shifting the design paradigm from single-process optimization to system-level power–performance–area (PPA) co-optimization, HI technology is now restructuring semiconductor ecosystems.

Current research efforts in this area include atomic-scale chip/wafer stacked bonding interfaces, thermal–mechanical reliability modeling, and AI-driven heterogeneous design automation frameworks [1,2]. These efforts aim to address the remaining challenges in chip/wafer-level warpage control and cross-domain signal integrity. The current wafer-to-wafer bonding technology features an interconnect Cu pitch size of around 1 µm. For AI applications, it is essential to push the Cu interconnect pitch to 400 nm and below [21]. Overlay control could be a challenging issue for the wafer bonding process, with suggestions that the overlay should be smaller than 100 nm to achieve a reasonable yield in mass production.

(b) Copper-based Interconnect Technology for Heterogeneous Integration

To achieve 3D stacking, a variety of 3D interconnect technologies have been developed, with interconnect pitches ranging from millimeters to less than 100 nm [5,22,23,24,25]. In older flip-chip packaging or face-to-face (F2F) integration technologies, solder bump and micro-bump (C4 bumps) interconnects were used. The pitch size of C4 bumps was usually greater than 50 μm, which is no longer suitable for the high interconnect density in modern computing architectures. A combination of copper pillars and solder caps in C2 bumps, which can further reduce the pitch width, could be a viable option.

Wafer-to-wafer hybrid bonding, particularly for AI SoC platforms, requires ultrafine pitch copper interconnects (<10 μm) with minimal interconnect parasitics, presenting significant challenges in various aspects of fabrication technology. Typical requirements include an interconnect density of over 10^6^ contacts/mm^2^, a contact resistance of 2–3 μΩ·cm, and a signal bandwidth of over 1 Tb/s/mm^2^ [5,21]. Conventional solder processes are no longer suitable; copper-based interconnects must be used. Copper is an ideal bonding material due to its high electrical and thermal conductivity, electromigration resistance, mechanical strength, and cost-effectiveness. Copper has a superior electrical conductivity of 5.96 × 10^7^ S/m, about five times larger than Pb-free solder, and a thermal conductivity of 401 W/m·K, which is eight times that of conventional Sn-Ag-Cu alloys [5]. However, the presence of copper pillars imposes significant requirements and challenges for assembly processes, particularly in terms of the submicron alignment precision required for ultrafine pitch interconnects.

The easiest way to achieve Cu-Cu bonding is thermal compression bonding (TCB). IBM demonstrated a 4 μm Cu bonded interconnect design and structure in 2006 [1,2]. Although the original copper-to-copper thermo-compression bonding has shown improvements in electrical performance and reliability compared to C2 bumps, the protruding copper structures still exhibit similar drawbacks as those found in conventional Cu-Cu bonding. Metal–dielectrics hybrid bonding was then proposed. Hybrid bonding technology, also called direct bond interconnect (DBI), was originally conceptualized in the mid-1980s and subsequently pioneered and industrialized in the early 2000s [1,2]. This technology can achieve ultrafine pitch and high-density interconnect integration through bonding metal pads and dielectric surfaces simultaneously at submicron alignment accuracy (<0.5 μm), revolutionizing chip packaging architectures and emerging as the critical enabling technology for heterogeneous integration and the chiplet-based systems in the post-Moore era.

The precise control of the surface topology, bonding nature, and surface morphology of dielectric materials, bonding mechanisms, and pre-bond and post-bond treatments is crucial for overlay control and interconnect pitch scaling. Additionally, dielectric breakdown is a critical factor affecting the yield of bonded wafers. Research indicates that unequally designed Cu pads exhibit higher dielectric breakdown strength compared to equally designed pads at small interconnect pitches. To achieve the necessary surface roughness and copper film uniformity across the wafer for producing Cu pads at the hundred-nanometer scale, a chemical mechanical polishing (CMP) step is required. Advances in surface plasma activation, passivation, and copper structure optimization show significant potential for achieving high-density, efficient heterogeneous integration. The integration of Cu/polymer hybrid bonding further enhances material compatibility and reduces process complexity.

The interfacial integrity and quality of Cu-Cu bonding are significantly influenced by surface planarization. Conventional processes typically require processing temperatures in the range of 300 to 400 °C under pressure to achieve sufficient atomic diffusion across the bonding interface. However, such a thermal budget exceeds the limitations of most back-end-of-line (BEOL) processes. Advanced pretreatment methods, like plasma activation or self-assembled monolayer passivation, have been developed to further reduce activation energy and achieve low-temperature processing. Additionally, hybrid bonding demands extremely low surface roughness to ensure a smooth and flat surface, necessitating the use of specialized CMP tools and abrasive slurries, which further increase the overall difficulty and cost. This paper systematically reviews various methods aimed at lowering process requirements and improving the overall quality of bumpless Cu-Cu bonding. These insights provide valuable guidance for the development of bumpless Cu-Cu bonding technology and heterogeneous integration.

## 2. Surface Plasma Activation

Surface plasma activation is a surface treatment technique used to improve surface adhesion. It relies on the strong adhesion force created when two atomically clean solid surfaces come into contact. These clean surfaces are usually achieved by dry processes, such as ion (Ar) beam bombardment or hydrogen radical irradiation [26,27]. Surface Activated Bonding (SAB) can be traced back to the direct bond interconnect (DBI) technology, initially proposed by Tong et al. [28]. This technique was initially proposed to (not limited to) the semiconductor industry for low-temperature and room-temperature bonding. The process mainly includes surface cleaning, planarization, surface activation (etching), terminating, and bonding under vacuum conditions. The wafers were left in a controlled environment for a specific period of time (typically hours to days) to allow the bonding interface to expand and merge spontaneously, and the effect of storage time and storage solution on bonding energy was investigated.

**Figure 2:**
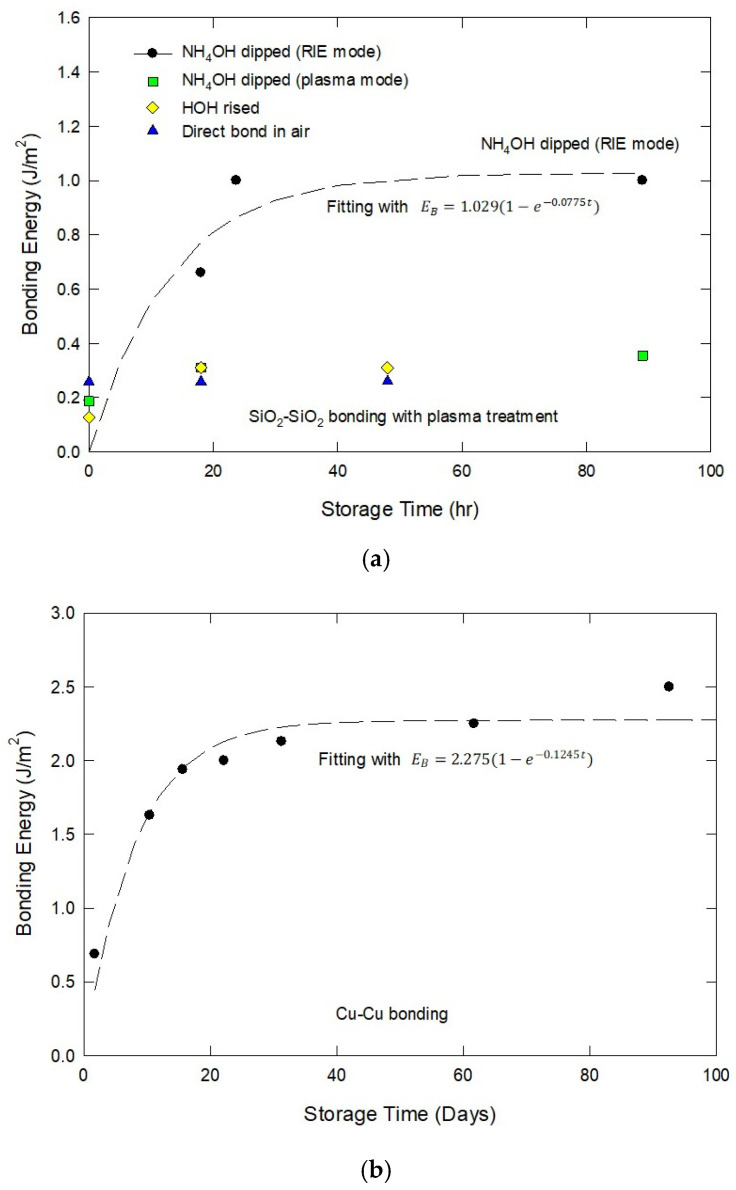
Typical bonding strength behavior as a function of storage time. (**a**) SiO_2_-SiO_2_ bonding, data taken from [28], and (**b**) Cu-Cu bonding, data taken from [29].

Figure 2a illustrates the bonding energy growth over storage time for SiO_2_-SiO_2_ bonding under various surface treatments, as reported by Tong et al. [28]. Their findings indicate that immersing SiO_2_ in NH_4_OH significantly enhances bonding energy. The bonding strength follows an exponential-saturation function, i.e., $E_{B}=a(1-e^{-bt})$, where *a* represents the maximum bonding strength. For Tong et al.’s data, *a* is 1.029 J/m^2^. A similar trend was observed in Cu-Cu bonding, as demonstrated by Lea Di Cioccio et al. [29] (see Figure 2b). Our model fitting estimates the maximum bonding strength at 2.275 J/m^2^. The initial weak bonding strength in Cu-Cu bonding is attributed to surface oxides, such as Cu(OH)_2_ or Cu_2_O. The subsequent increase in bond strength results from the dissolution of these oxides, a process governed by the oxygen diffusion rate across the copper film, which conforms to an exponential saturation growth function. For SiO_2_-SiO_2_ bonding, the observed bonding strength improvement is likely due to the release of hydroxyl groups adsorbed onto the SiO_2_ surfaces, facilitating stronger adhesion over time.

**Figure 3:**
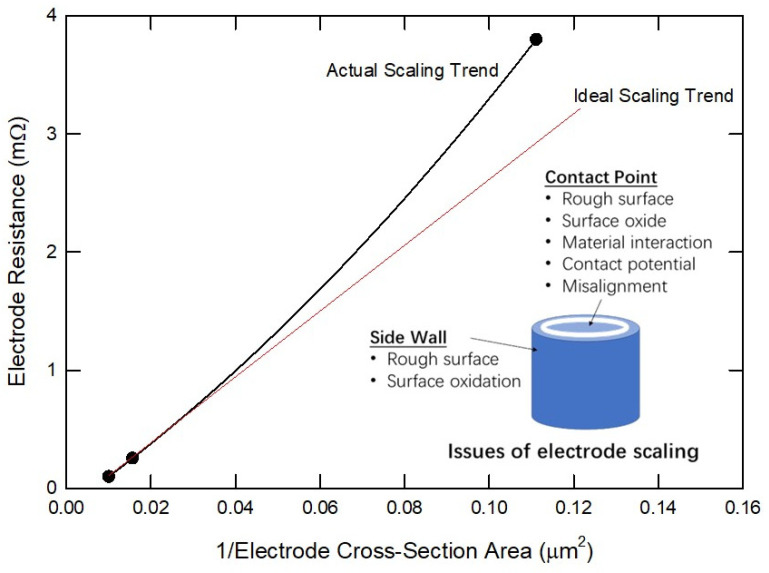
Plot of the contact resistance of bonded Cu pads corresponding to the diameters of electrodes. The markers are data taken from [6], and the red lines represent the calculated resistance based on Ohm’s law.

The possibility of surface-activated bonding for ultrafine pitch Cu interconnection has also been studied over the years [6,26]. SAB, based on the flip-chip bonder with 10 μm bonding pitch, was realized in 2006 [6]. In that study, the alignment error was also kept very small, which was less than ±1 μm. Figure 3 illustrates the measured electrode resistance as a function of electrode size. As the contact size or electrode size decreases, non-ideal effects—including contact potential, surface scattering, and surface oxidation—become increasingly pronounced. Consequently, the electrode resistance deviates from the linear curve dictated by Ohm’s law.

**Figure 4:**
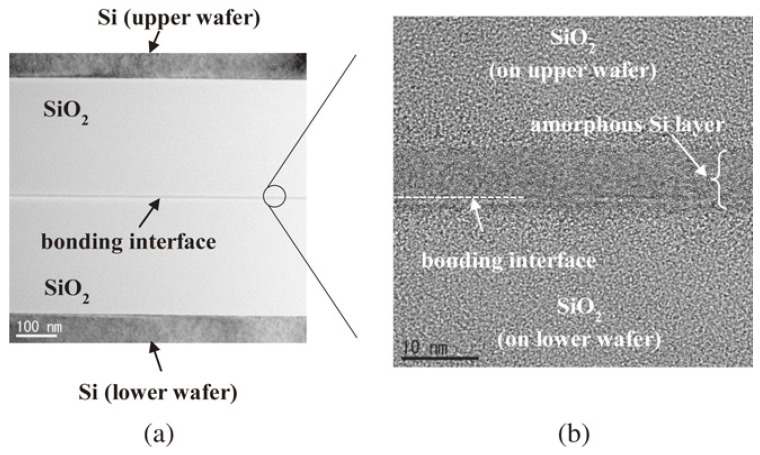
TEM cross-sectional image of a Si-containing SiO_2_/SiO_2_ bonding interface: (**a**) low magnification and (**b**) high resolution. Voids are not observed at the interface, but there is a layer of amorphous Si above and below the bonding surface, caused by different Ar fast atom irradiation to the upper and lower wafers Reproduced with permission from [30], Jpn. J. Appl. Phys.; published by IOP, 2016.

**Figure 5:**
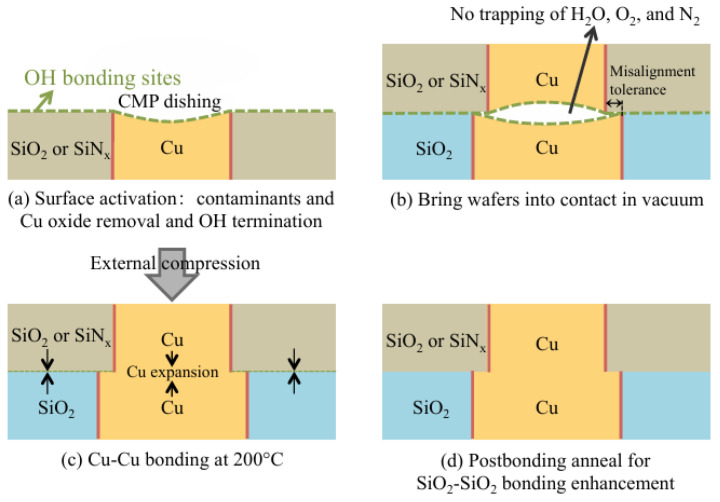
Schematic of common hybrid bonding steps with the combined SAB method Reproduced with permission from [31], ECS Trans.; published by IOP, 2016.

**Figure 6:**
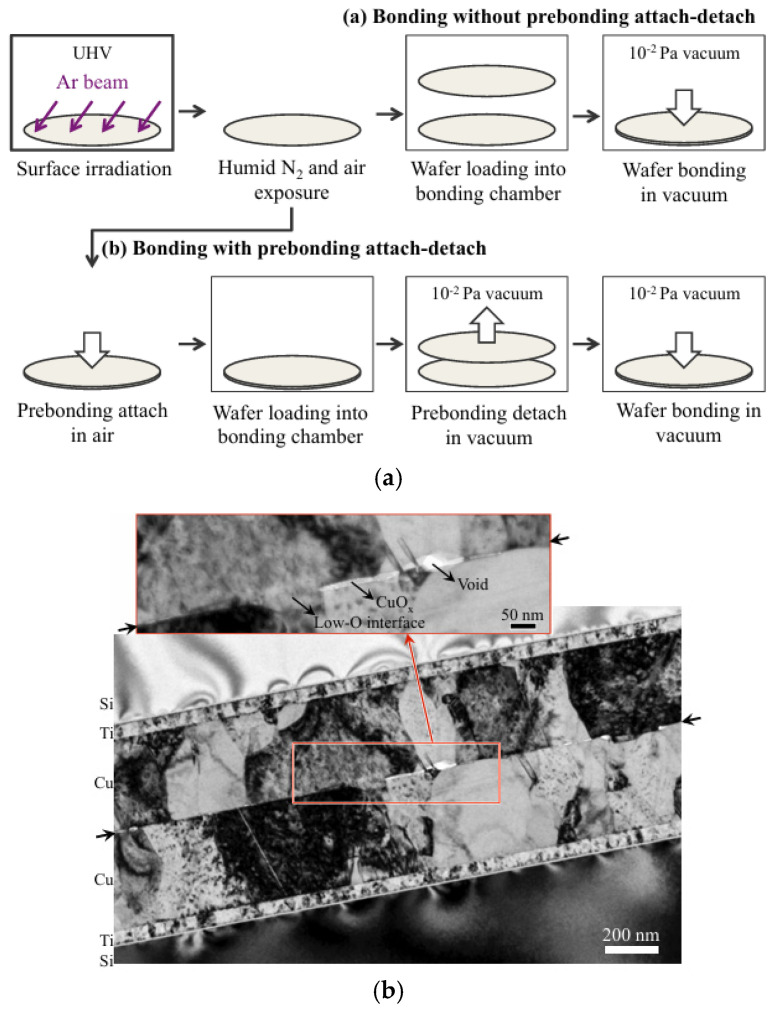
(**a**) Process flow of combined SAB without and with prebonding attach–detach. (**b**) TEM image of the Cu-Cu bonding interface after postbonding annealing using the combined SAB method, with a few voids and CuO_x_ Reproduced with permission from [31], ECS Trans.; published by IOP, 2016.

**Figure 7:**
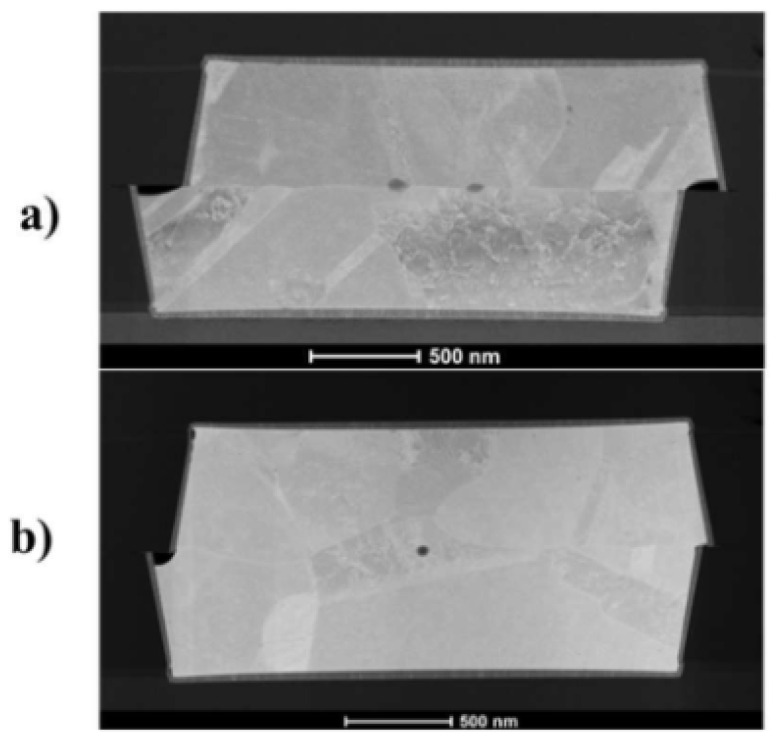
TEM cross-sectional images of SAB Cu pads bonded at room temperature: (**a**) SAB without annealing; (**b**) SAB and annealed at 150 °C for 2 h Reproduced with permission from [32], ECS Trans.; published by IOP, 2023.

**Figure 8:**
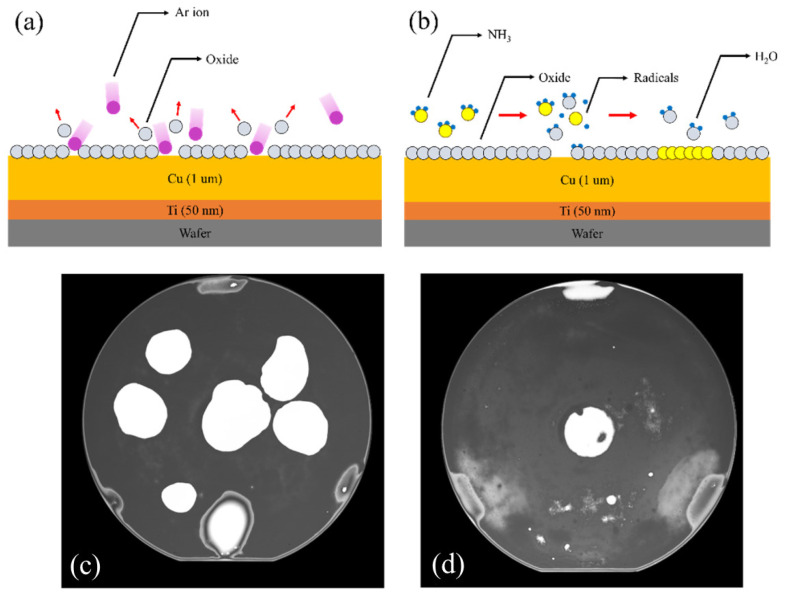
Schematic and SAM data of different plasma surface treatments: (**a**) Ar plasma surface treatment; (**b**) NH_3_ plasma surface treatment. The corresponding results are depicted in (**c**) and (**d**), respectively. Reproduced with permission from [17], Coatings, published by MDPI, 2024.

Utsumi later introduced silicon ultrathin films on SiO_2_ surfaces before SAB to facilitate dielectric bonding [30]. Ar plasma was used to activate the surfaces, resulting in a bonding strength of 25 MPa in tensile testing [30]. Figure 4 shows the SEM cross-sectional image of the bonding interface containing the Si layer formed by deposition. The research provides a novel low-temperature method for dielectric bonding as well as Cu/dielectric hybrid bonding in heterogeneous integration applications. A conventional hybrid bonding flow is employed, as shown in Figure 5. The combined optimization includes extra Si atoms in irradiation, which increases oxygen vacancies on the SiO_2_ surface and promotes OH absorption when bonding. Meanwhile, the prebonding attach–detach process makes hydroxylation more effective and creates more OH sites on the SiO_2_ surface. Soon after, He et al. combined Si-containing Ar beam irradiation and a prebonding attach–detach procedure, achieving high-quality and 200 °C hybrid bonding [31] (Figure 6a). The results after hydrophilic bonding are displayed in Figure 6b. Renaud et al. further realized SAB hybrid bonding at room temperature with a pitch width of only 5 μm and a thermal budget as low as 150 °C (annealing at 150 °C) [32]. The key to sufficiently high adherence is the precise control of Cu recess depth during surface activation etching, since the thermal expansion of Cu at low-temperature annealing will not be sufficient to fill the gaps [32]. The bonding results are shown in Figure 7. With NH_3_ plasma surface treatment, as illustrated in the upper part of Figure 8, Jeon and Hong demonstrated a superior efficacy in improving surface smoothness and thus the bonding quality [17]. Comparative analysis of scanning acoustic microscopy (SAM) data of the Cu surface is shown in the lower part of Figure 8. Surface plasma activation is virtually indispensable in low-temperature and high-quality fine-pitch hybrid bonding. However, plasma activation requires an ultra-high vacuum environment and a specific CMP to create an extremely low roughness surface, which expands the production cycle and adds significantly to the cost.

## 3. Passivation

**Figure 9:**
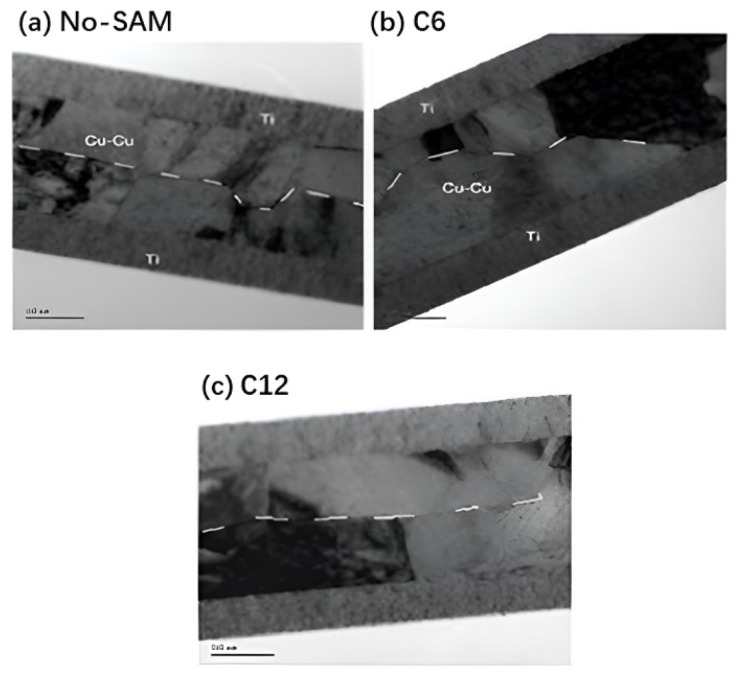
TEM comparison of the Cu-Cu bonding interface without and with a passivation layer composed of linear chain alkane-thiol with six (C6) and twelve (C12) carbon atoms, respectively: (**a**) without SAM passivation, (**b**) with C6 SAM passivation, and (**c**) with C12 SAM passivation. Dashed lines indicate the interfaces. Reproduced with permission from [34], ECS Trans.; published by IOP, 2012.

If the substrate is exposed to the atmospheric environment before bonding, an oxide layer on the Cu surface will inevitably form, which is one of the significant problems limiting the bonding quality and thermal budget. Passivation has been widely used in Cu-Cu bonding to prevent the oxidation of Cu by inhibiting the interaction between the surface and oxygen. Additionally, when a rare metal with excellent electrical and physical properties is used as a passivation material, it could enhance the conductivity of the interconnect and improve its mechanical stability. The alkane-thiol self-assembled monolayer (SAM) passivation technique has been explored for Cu-Cu bonding [33,34]. This method is non-corrosive and eliminates the need for high vacuum environments. The Cu layer exhibits significant interdiffusion and grain growth, as shown in Figure 9, providing a promising solution for low-temperature bonding [33,34]. However, thermal or mechanical desorption is required before bonding, and SAM passivation layers may leave potential surface residues after desorption.

**Figure 10:**
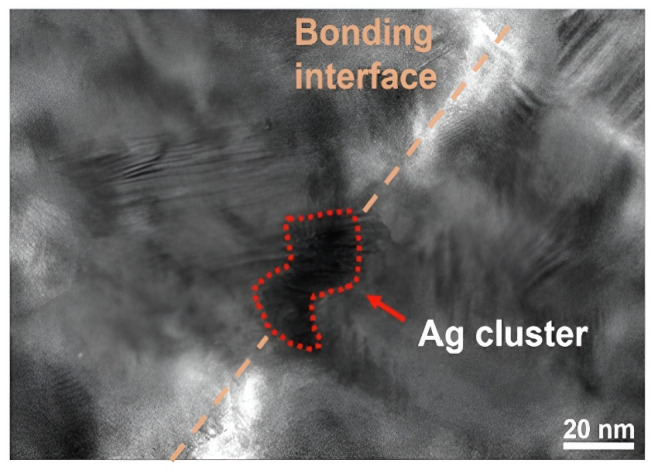
TEM image of the ultrathin cluster-Ag passivation structure bonded at 150 °C. The contacted Ag passivation layers with fragment metal grain form the cluster structure, while Cu diffuses through the cluster gaps and fills up the voids. Reproduced with permission from [37], Appl. Surf. Sci.; published by Elsevier, 2023.

**Figure 11:**
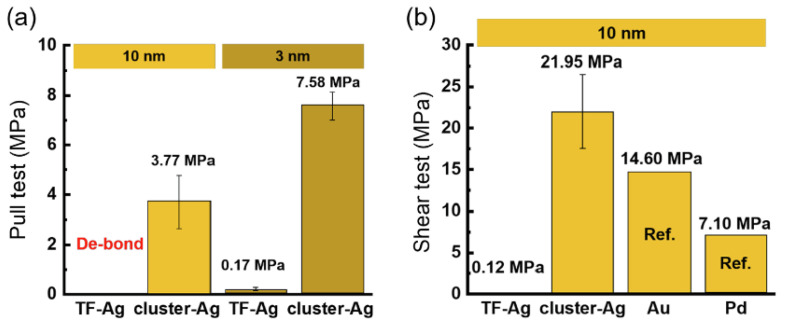
Mechanical strength test plots of Ag, Au, Pd, and cluster-Ag passivation layers at 10 nm and 3 nm: (**a**) pull test; (**b**) shear test. The strength of the wafer bonded with 10 nm silver passivation was too low and prematurely debonded in the pull test. Reproduced with permission from [37], Appl. Surf. Sci.; published by Elsevier, 2023

**Figure 12:**
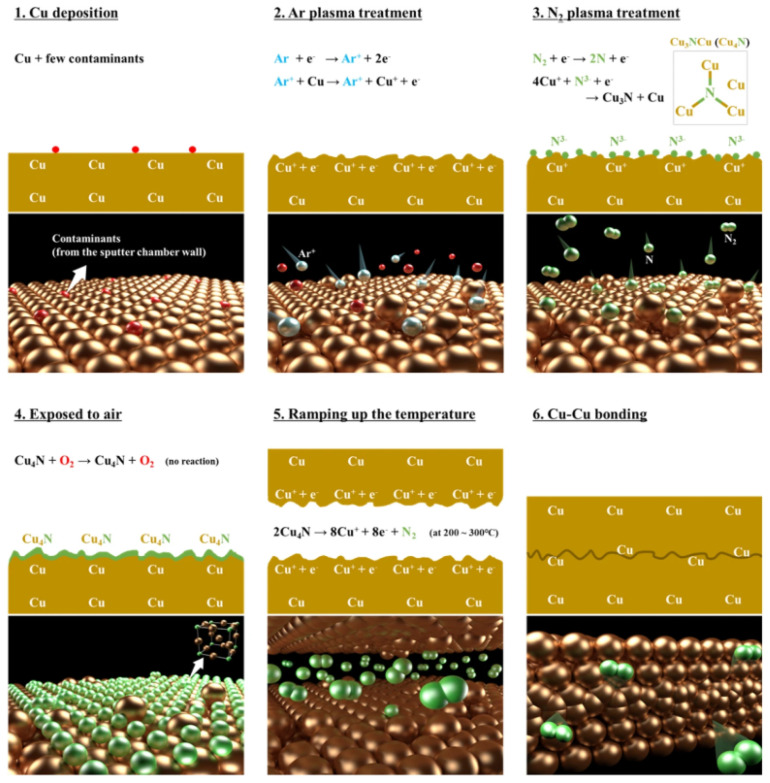
Low temperature Cu-Cu bonding mechanism of the two-step Ar/N_2_ plasma process. Reproduced with permission from [38], Sci. Rep.; published by Nature, 2020.

**Figure 13:**
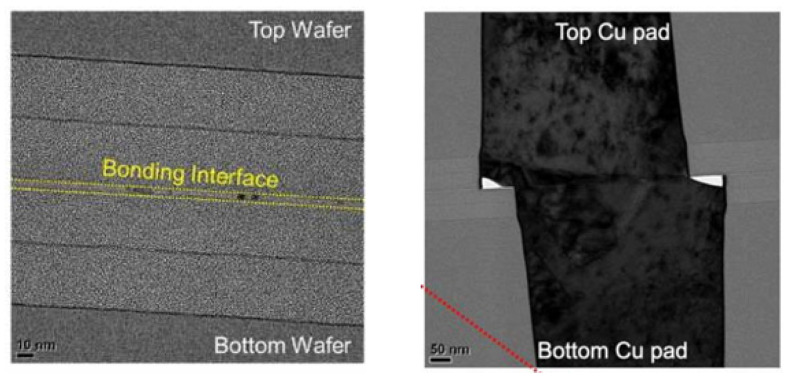
TEM images of SiCN-SiCN (**Left**) and Cu-Cu (**Right**) bonding interfaces with N_2_ plasma treatment by contamination-free nitrogen passivation strategy. Reproduced with permission from [39], ECTC, published by IEEE, 2023.

Passivation with metal layers was also attempted. It was found that Pd, Au [35,36], and Ag [37] can be used as the passivation layer for Cu-Cu bonding. Among the materials for the passivation layer, those with larger lattice constants and favorable diffusion of copper atoms, as well as thinner passivation layers, tend to have higher strength after bonding. In addition, the resistivity of the passivation metal is also a factor worth considering. Silver clusters as a passivation layer have also been studied [37], demonstrating better bonding quality. Silver clusters are formed by a silver particle layer prepared by ultra-low energy sputtering (~0.01 W/cm^2^), which aggregate into silver clusters during bonding. During aggregation, space is created between cluster gaps, allowing two Cu layers to contact directly (as shown in Figure 10), resulting in a higher bonding quality than previous bonding methods that rely on the diffusion of Cu atoms through the passivation layer. This method achieved notable results in pull and shear tests, with bonding strengths of 3.77 MPa and 21.95 MPa, respectively, at a low bonding temperature of 150 °C. Figure 11 demonstrates the comparison of the bonding strength of Ag, Au, and Pd as passivation layers. Park et al. recently reported an alternative compound passivation material [38,39]. They used Cu_4_N as the passivation layer. This material has the advantage of being non-reactive with air at room temperature while leaving the substrate as N_2_ when bonding at 200–300 °C. This passivation technique typically consists of two plasma treatment steps: after argon plasma activation to remove native oxide and contaminants, the N_2_ plasma treatment is carried out to generate a passivation layer by the reaction of N and Cu. The more detailed steps and schematic of microscale surface reactions are shown in Figure 12. Hahn et al. performed Cu/SiCN hybrid bonding with N_2_ plasma surface treatment [39]. This technique reduces the Cu pad pitches down to 0.4 μm. It was noted that conventional plasma treatments could lead to Cu atoms being knocked out from the Cu surface, which is gradually becoming non-negligible in submicron pitch interconnects, as copper re-sputtering reduces the distances between Cu pads and even creates conductive pathways on the dielectric surface. At the same time, this can contaminate the bonding environment and reduce the chamber lifetime. To address the issue, reactive molecular dynamics (MD) simulations were carried out in the research to determine the parameters of plasma treatment, and the optimized plasma treatment can meet the contamination-free standard [39]. TEM images of the SiCN and Cu bonded interface are displayed in Figure 13.

## 4. Copper Geometry Optimization

**Figure 14:**
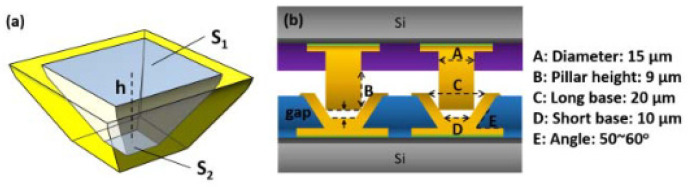
(**a**) Three-dimensional schematic and (**b**) cross-sectional schematic of the pillar–concave Cu structure for high-pressure Cu-Cu bonding. Reproduced with permission from [41], IEEE Trans. Compon. Pack. Manuf. Technol.; published by IEEE, 2020.

**Figure 15:**
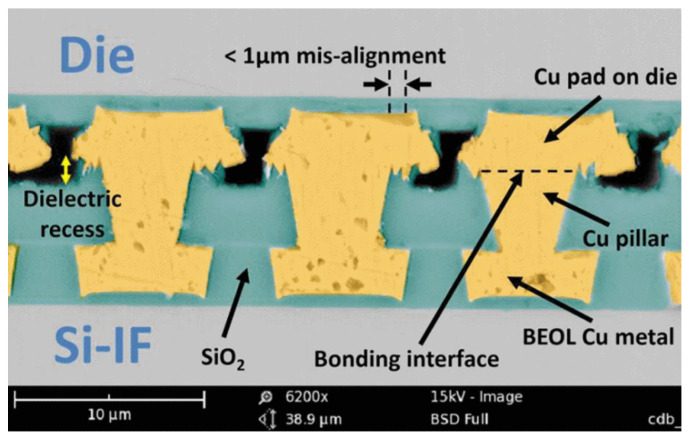
SEM image of high-throughput two-stage TCB with protruding Cu pads. Reproduced with permission [43], ECTC, published by IEEE, 2023.

In traditional TCB bonding, high pressure is applied to intensify the interdiffusion of Cu atoms and promote the breakdown of surface oxide, thereby achieving a more direct and robust bonding interface [40]. Optimizing the geometry of the bonding interface facilitates a more rational distribution of bonding pressure and allows for more effective deformation. Chou et al. investigated a pillar–concave Cu structure (as shown in Figure 14) that is able to perform high-quality bonding with a low thermal budget (150 °C) and high roughness of the bonding interface [41]. The mechanism behind this is that Cu pillars deform plastically and generate lattice dislocations inside the material when in contact with the angled concave surface under strong external pressure (>500 MPa). During deformation, sliding and friction of dislocated surfaces generate internal energy, which promotes the interdiffusion and regrowth of Cu atoms [42]. Another high-throughput bonding scheme with the structure of Cu pads protruding over the dielectric surface was proposed recently [43]. The method has no dielectric bonding process and only consists of two steps, which are Cu tacking and annealing. Achieving high alignment precision is critical in this streamlined bonding scheme because the quality of the substrate depends on the bonding strength of Cu pads. The SEM image of the Cu bonding interface presented in Figure 15 demonstrates excellent bonding quality achieved with low acceptable misalignment. High-pressure bonding with an optimized structure tends to have the advantages of short process flow, low operation temperature, low cost (CMP-free), and high throughput, but relatively high bonding pressure (500 MPa and 50 MPa, respectively, in the literature above) limits the scope of applications to some extent.

**Figure 16:**
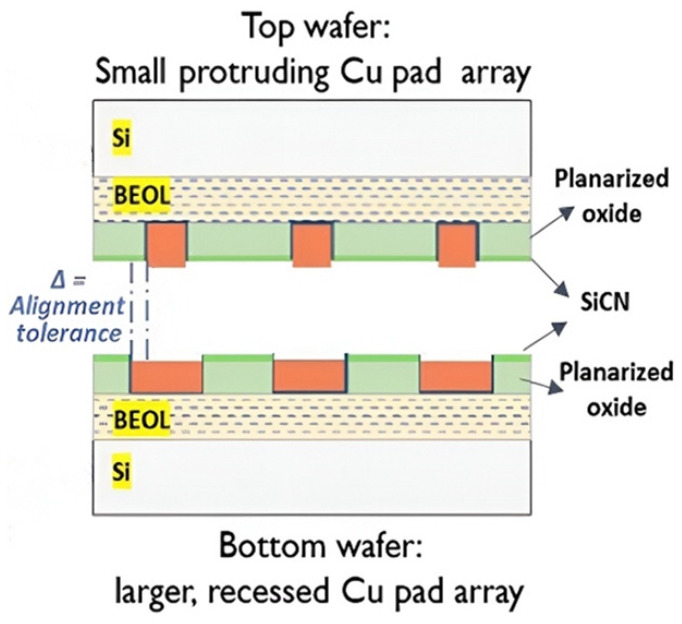
Schematic of Cu/SiCN hybrid wafer-to-wafer bonding with a protruding/recessed Cu pad array. Reproduced with permission from [44], ECTC, published by IEEE, 2020.

**Figure 17:**
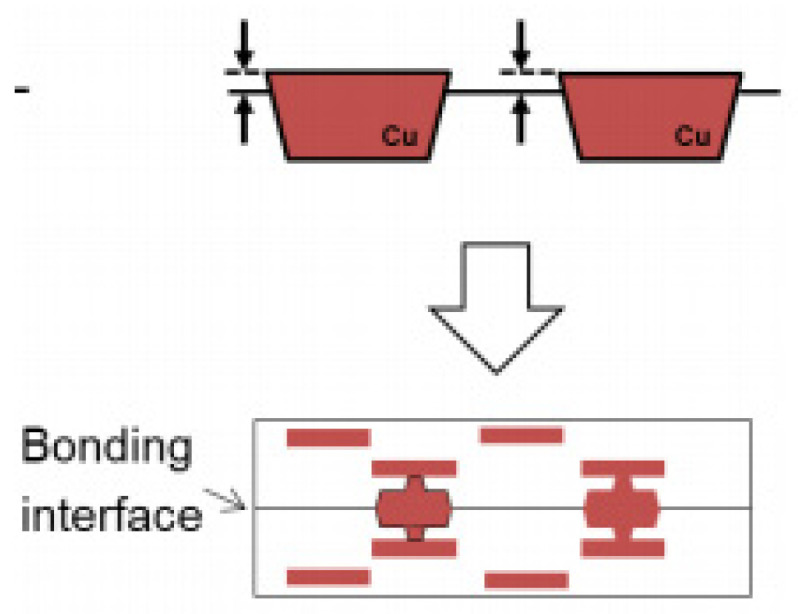
Schematic of ultrafine pitch hybrid bonding with a protruding Cu pad structure. Reproduced with permission from [45], ECTC, published by IEEE, 2022.

**Figure 18:**
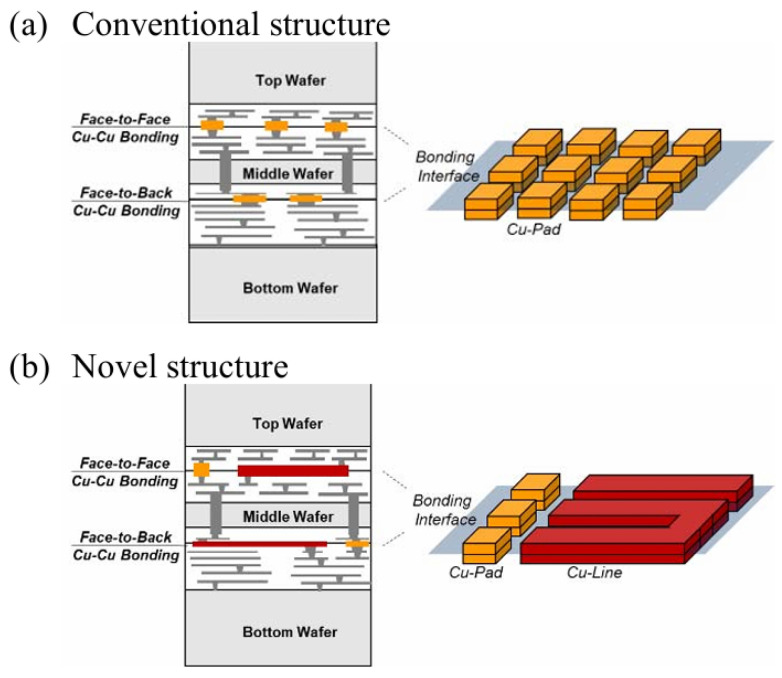
Cross-sectional image and schematic of (**a**) the conventional Cu-Cu hybrid bonding structure and (**b**) a Cu-Cu wiring structure in face-to-face and face-to-back bonding. Reproduced with permission from [46], ECTC, published by IEEE, 2023.

**Figure 19:**
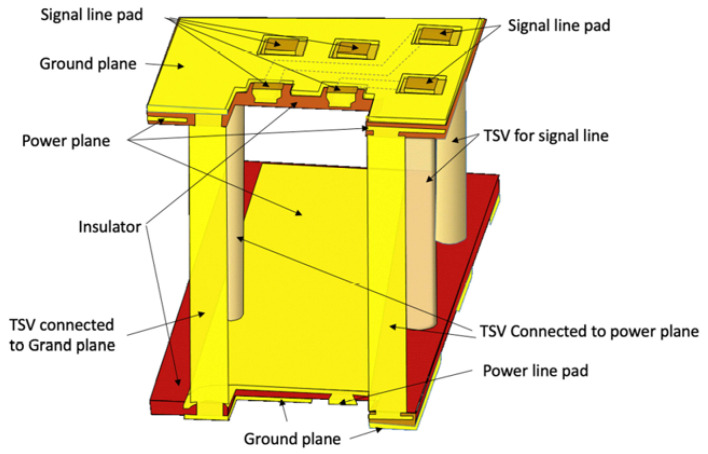
An example of an AC3D interconnect. A small insulation layer surrounds the area around the Cu electrode on the wafers, while the rest is covered with Cu solid layers. Reproduced with permission from [47], ECS Trans.; published by IOP, 2023.

It is worth mentioning that, in addition to high-pressure bonding, the method of Cu topography control has also been widely used in fine-pitch hybrid bonding. IMEC introduced a Cu/SiCN bonding technology for direct wafer stacking that achieves interconnect pitches as small as 1 μm. In this method, one bonding wafer features Cu pillars that protrude slightly above the surface, while the corresponding bonding wafer has larger-area Cu pads recessed slightly below the surface, as shown in Figure 16. This complementary design effectively addresses the impact of overlay misalignments during the bonding process [44]. Sony conducted research on the wet process, the electrical chemical deposition (ECD) process, and the CMP process to stably fabricate slightly protruding Cu pillar structures (Figure 17). These structures are suitable for use in face-to-face and face-to-back hybrid bonding for 3D stacking technologies [45]. Building upon that approach, Sony introduced another Cu-Cu wiring technology that provides greater flexibility in circuit design. This innovation leverages Sony’s advancements in the CMP process, which enables multiple Cu patterns to maintain planar surfaces and ensures uniform contact and reliable bonding between layers in 3D stacking. Cu-Cu wiring can replace a part of Cu wires in the traditional internal interconnect layer and has lower electrical resistance due to the increased volume of Cu from two Cu pads bonding together, which is essential for high-speed signal transmission and efficient power delivery [46]. Figure 18 illustrates a comparison of Cu-Cu wiring and conventional Cu pad structures. In a further step, all-Cu 3D (AC3D) interconnect was introduced [47]. This process reduces the area of the insulating layer around Cu electrodes (see Figure 19). Such Cu solid layers form the ground plane, supply plane, and paired layers and help dissipate heat when connected to vias. Although some Cu polishing or electrode configuration issues remain unsolved, AC3D is very promising for the future of 3D integration, owing to its simple Cu-Cu bonding process.

**Figure 20:**
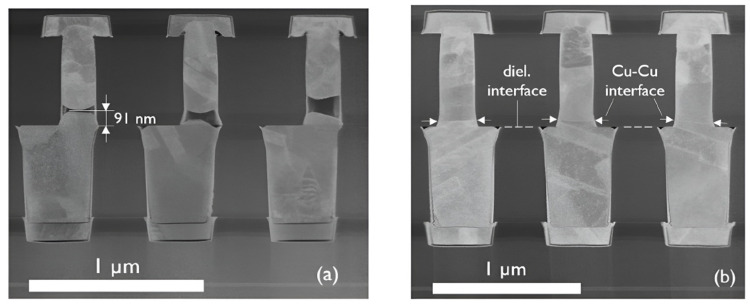
TEM images of fine pitch bonding with unequaled pad sizes, revealing the “Bulge-Out” phenomenon. (**a**) Defective connections caused by excessively recessed Cu pads. (**b**) Excellent connections with proper Cu pad height. Reproduced with permission from [48], ECTC, published by IEEE, 2023.

In the case of fine-pitch bonding with unequal pad sizes on bonding wafers, the IMEC group observed a phenomenon called “Bulge-Out” [48]. As depicted in Figure 20a, when small pads that are excessively recessed due to CMP issues are bonded to large pads, Cu protrudes from the bottom pad and extends into the cavity of the upper pad. This spontaneous protrusion phenomenon is very conducive to full contact between Cu pads, and Figure 20b shows the excellent bonding quality achieved with the existence of “Bulge-Out” after the correct CMP process. This mechanism was attributed to the Cu surface diffusion, and it was estimated that the best bonding result could be achieved when the ratio of the two pads’ area is 2:1. This discovery greatly relaxes the CMP process and holds significant potential for extending hybrid bonding to submicron pitch.

## 5. Copper Microstructure Optimization

**Figure 21:**
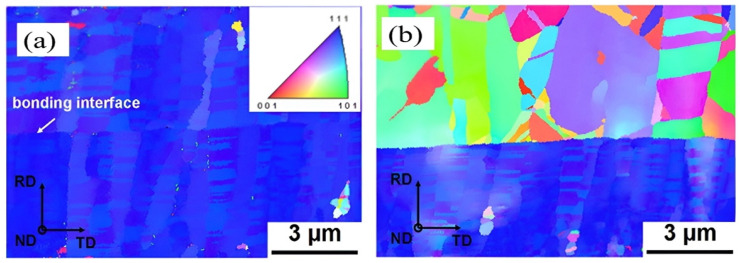
EBSD orientation image of optimized-orientation Cu pads (**a**) and random-orientation Cu pads bonded at 200 °C for 30 min (**b**). Reproduced with permission from [49], Scr. Mater., published by Elsevier, 2014.

**Figure 22:**
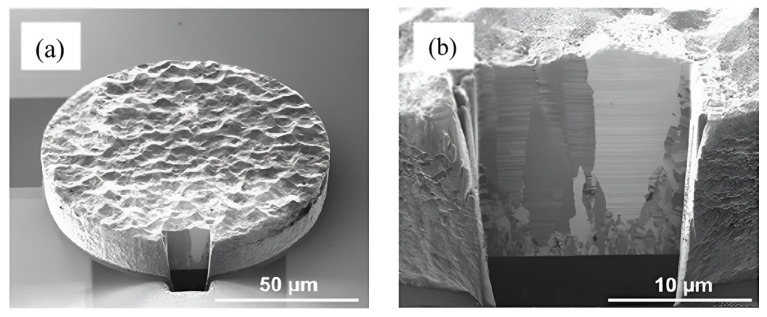
(**a**) FIB tilt-view of a nanotwinned Cu pad. (**b**) Enlarged view of a nanotwinned Cu pad at the cut surface by ion etching. Reproduced with permission from [52], Cryst. Growth Des.; published by ACS, 2012.

**Figure 23:**
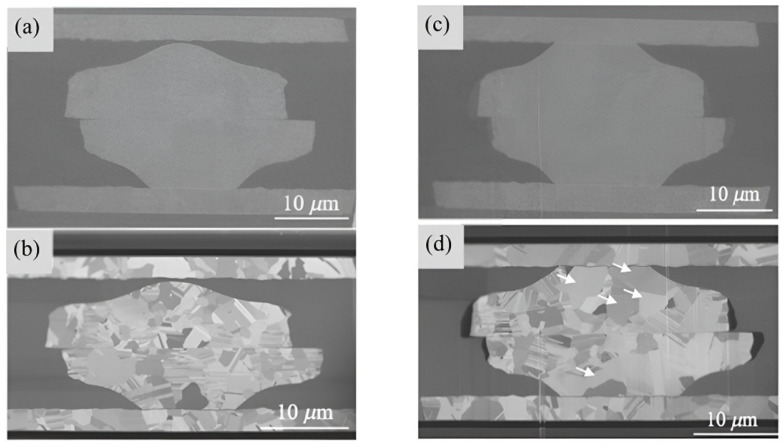
Elctron and ion images of a <111>-oriented nt-Cu bonding interface. (**a**) electron image and (**b**) ion image for after 10 s bonding; (**c**) electron image and (**d**) ion image after 60 s bonding. Arrows indicate the microscope changes as compared with the 10 s bonding. Reproduced with permission from [53], Jpn. J. Appl. Phys.; published by IOP, 2020.

Copper film, as-deposited, is in polycrystalline form. Even with thermal treatment, the grain size is still not large enough to extend continuously from one side to the other of the interconnects. A comparison between pads of optimized and untreated orientation after bonding was given by Liu et al. (see Figure 21) [49]. This discontinuity causes a reduction in the electrical conductivity of the interconnects and also reduces their mechanical stability, increasing the risk of cracking or delamination. Another advantage of optimizing the microstructure of copper is that, within temperatures of 120 to 250 °C, which is an acceptable temperature range for heterogeneous integrated devices in BEOL processes, the (111)-oriented nanotwinned copper (nt-Cu) exhibits the fastest surface diffusivity and the lowest oxidation rate [50]. The bonding mechanism of (111)-oriented nt-Cu is surface creep, which is atomic diffusion under a stress gradient under thermal compression. A kinetic model was developed by Chen et al. [51]. Nanotwinned Cu pads were then fabricated by dc electroplating on a Cu seed layer with a (111) preferred orientation. Figure 22 illustrates the EBSD orientation image of the Cu pads [52]. It was further found that direct bonding can be completed within 10 s by using (111)-oriented nt-Cu bumps in a N_2_ bonding environment [53]. This instant bonding has an advantage in mass production, but there still exists a noticeable interface between bonding pads, even if the bonding time is extended to 60 s (see Figure 23). Low-temperature (200 °C) and low-pressure (1.06 MPa) wafer-to-wafer hybrid bonding using (111)-oriented nt-Cu, with the lowest contact resistance (1.2 × 10⁻^9^ Ω·cm^2^) among the bonding methods, was reported [54]. The use of (111) surface nt-Cu not only leads to a very low resistance but also, due to the high hardness of nt-Cu, the height and shape of copper can be more easily controlled by CMP, further improving the quality of hybrid bonding [54].

**Figure 24:**
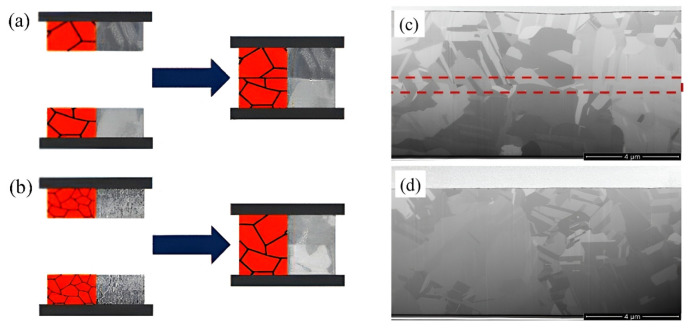
(**a**) A schematic diagram of bonding after grain growth has stopped (**b**). A schematic diagram of bonding at small grains (**c**) (see the region highlighted with red dashed lines. SEM image of the interface bonded after grain growth has stopped (**d**). SEM image of the interface bonded at small grains. Reproduced with permission from [55], ECTC, published by IEEE, 2021.

**Figure 25:**
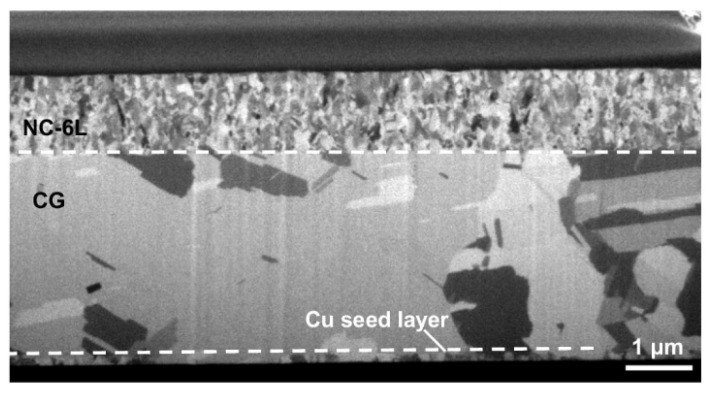
SIM image of the as-prepared double layer structure (high-impurity nanocrystalline layer and coarse-grained layer); the original boundary of the two layers is marked by the white dashed line. Reproduced with permission from [57], Nat. Commun.; published by Nature, 2024.

Apart from nt-Cu, the potential of nanocrystalline copper bonding was also investigated. It was found that copper fabricated through electrochemical deposition exhibits a self-annealing phenomenon; that is, copper grains grow larger at room temperature from being deposited up to a certain size [55,56]. The duration of this grain growth varies depending on the electrolyte used during deposition. To further explore this, a comparative experiment simulating bonding was conducted. A second layer of copper was deposited onto freshly deposited copper and where grain growth had ceased, using the same process. These samples were then annealed at 200 °C for one hour to compare the ability of grain growth across the interface. The results demonstrated that freshly deposited copper has a higher interfacial quality [56]. A schematic diagram and SEM images illustrating these findings are shown in Figure 24. A process allowing the metastable microstructure to be maintained for 48 h, or even 120 h with additional post-processing, was proposed recently. It makes the process more flexible [56]. Nanocrystalline Cu is typically fabricated with additive overdosing and will introduce elevated impurity co-deposition. This problem may lead to void formation, incomplete grain growth, and reliability issues like electromigration. A double-layer structure is developed, integrating a coarse-grained (CG) layer beneath the nanocrystalline layer. The CG layer functions to facilitate impurity diffusion from the nanocrystalline layer and thus suppress microstructural defects and impurity aggregation [57]. Figure 25 shows a SIM image of the double-layer structure after fabrication.

## 6. Cu/Dielectric Hybrid Bonding

The advantage of hybrid bonding technology lies in its ability to achieve high-density heterogeneous chip stacking, meeting the demand for higher power, performance, and cost-effectiveness [58,59]. As the application domain expands, the future may see more advanced implementations of hybrid bonding, such as stacking logic with logic and memory with logic. This will drive the further development of 3D system integration design, requiring the next generation of bonding devices to meet more stringent standards in terms of overlap accuracy, thus achieving higher production efficiency and reliability.

Cu/oxide and Cu/polymer hybrid bonding technology, as the core enabling technology of 3D heterogeneous integration in the post-Moore era, has experienced rapid development from process optimization to large-scale production in recent years. Cu/SiO_2_ hybrid bonding technology can achieve ultra-high density (>1 × 10^6^/mm^2^), submicron alignment (<0.5 μm), and excellent thermo-mechanical reliability through the covalent bonding of copper interconnects to silica media, which has become the core solution for 3D heterogeneous integration. Its core advantages are low interface resistance, high temperature stability, and high compatibility with CMOS technology, which support the performance leap of high-performance computing (HPC), high bandwidth memory (HBM), and advanced packaging. Up to now, the world’s leading semiconductor companies and research institutions have made a series of breakthroughs in three directions: interconnection density enhancement, thermo-mechanical reliability enhancement, and low-temperature process development. TSMC took the lead in realizing the heterogeneous integration of advanced node logic Chips and CoWoS (Chip-on-Wafer-on-Substrate) packaging in its SoIC (System on Integrated Chips) platform [11]. By optimizing the morphology of copper pillars and the hybrid bonding process, the interconnect spacing is reduced to below 1 μm, the bond thickness is limited to less than 10 μm, which supports a high bandwidth memory for HPC applications.

**Figure 26:**
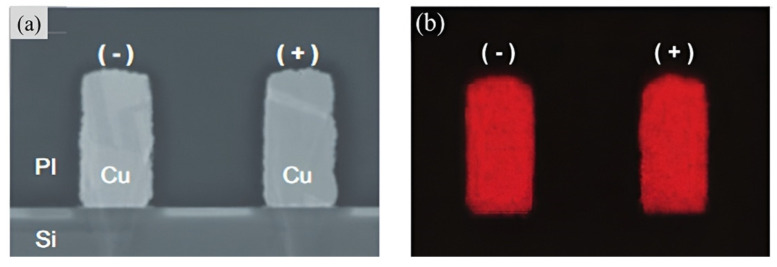
Result of biased HAST with the new PI test vehicle of 2 μm L/S. (**a**) Cross-sectional SEM image in a freely selected area and (**b**) EDX result. Reproduced with permission from [61], ECTC, published by IEEE, 2021.

Copper/polymer hybrid bonding has emerged as a promising interconnect solution in advanced packaging, leveraging the polymer’s intrinsic low dielectric constant and robust copper diffusion barrier properties to simultaneously mitigate RC signal attenuation and enhance interconnect reliability in high-density configurations. Compared to conventional Cu/SiO_2_ hybrid bonding, Cu/polymer architectures demonstrate superior compatibility with particle-contaminated processes due to the polymer’s inherent mechanical compliance, which locally deforms to encapsulate contaminants rather than inducing bonding defects [60]. Furthermore, the polymer’s elevated coefficient of thermal expansion (CTE) facilitates void-free interfaces through enhanced conformal filling during thermal processing, while its lower elastic modulus can mitigate CTE mismatch and wafer warpage. However, Cu/polymer surfaces face critical challenges in achieving planarization during the chemical mechanical polishing (CMP) process, primarily due to mechanical property disparities, such as modulus mismatch and the polymer’s rheological behavior under shear stress. A novel photosensitive polyimide (PI) and thermal compression bonding process was proposed [61]. This specialized PI is compatible with typical CMP processes and does not require any special pretreatment in the pre-bond process. The new PI-based technique has demonstrated good reliability, with no Cu diffusion observed during reliability tests, as shown in Figure 26.

Due to the high acid resistance of polymers, wet pretreatment is often used to remove oxide from the surface of copper pads. A study examined the effect of different acidic aqueous treatments on bonding quality and concluded that pretreatment with citric or ascorbic acid can significantly improve the bonding yield of copper pads [62]. To further enhance the coplanarity of copper/polymer surfaces, Jang et al. proposed a flying cut method to replace the CMP process, coupled with a surface cleaning process before bonding to remove the generated residues [63]. A perfect polyimide bonding surface was obtained after bonding, but seams were observed on the Cu-Cu interface, which may be attributed to the softer mechanical properties of the polymer and thus better resistance to contamination. Optimization of the cleaning procedure after a flying cut or Cu surface activation may permit a better bonding interface and broader application of this technology [63].

It is noted that the present Cu/polymer hybrid bonding technology is limited by the nature of the polymer material and process, such as a relatively high glass transition temperature (Tg ~ 250 °C), high interfacial resistance (2–5 Ω·μm^2^), and hygroscopic expansion issue, which would lead to challenges in long-term reliability. At present, it is mainly applied to 5G RF modules, wearable sensors, silicon optical engines, and other medium- and low-power scenarios. With the development and evolution of hybrid technology, researchers and engineers can choose different technology paths and innovative technologies for scene adaptation according to different application scenarios and future product requirements. Cu/oxide will continue to evolve to submicron interconnects (<0.5 μm spacing) and a low-temperature activation process (plasma-assisted bonding ≤ 250 °C) to support AI accelerators and memory and computing integrated architectures. On the other hand, Cu/polymer has gradually penetrated into emerging fields, such as heterogeneous integrated photonic (CPO) and bio-MEMS through the development of low moisture absorption nanocomposites and submicron alignment technology (<0.3 μm) [55]. Cu/oxide dominates the 3D integration of high-power chips, while Cu/polymer covers low-power consumer electronics and specialty packaging. The two-hybrid bonding interface techniques achieve system-level performance and cost optimization through heterogeneous layering by combination with low-level oxide bonding and upper polymer interconnect, which drives the diversification of heterogeneous integration technologies in the post-Moore era.

## 7. Conclusions

In summary, while Moore’s Law is expected to persist for at least another decade in terms of chip density and the number of transistors, the technology driving this progress will undergo significant transformations. Traditional gate length scaling has reached its limits, and future advancements will heavily depend on vertical stacking and heterogeneous integration. Technologies such as CFET and 3D monolithic stacking of various functional modules, particularly memory modules, will be pivotal. These advancements hold the potential to achieve trillion-level integration in the coming decades, driven by their flexibility, scalability, and integration efficiency. Additionally, 3D stacking technology facilitates the integration of non-digital circuits, incorporating sensors and analog/RF functionalities through silicon vias (TSVs).

This work highlighted recent advancements in the copper-to-copper (Cu-Cu) bonding technology for system-on-chip (SoC), wafer-to-wafer, and heterogeneous integration applications. Particular attention was given to innovative processes, such as surface plasma activation, passivation, and copper structure optimization. Additionally, the integration of Cu/polymer hybrid bonding enhances material compatibility and reduces process complexity. Current wafer-to-wafer bonding technology, with an interconnect Cu pitch size of around 1 µm, needs to be reduced to 400 nm and below for trillion-level integration and AI applications. With this connection, we also addressed the scaling and overlay control issues associated with Cu-Cu bonding. The impact of these advancements is profound, as they address critical bottlenecks in interconnect scaling and pave the way for high-density, efficient heterogeneous integration, and SoC applications. Table 1 lists the methods of bonding discussed in this paper and provides key factors, such as pitch width, bonding conditions, and bonding quality achieved.

As previously mentioned, scaling down the bump dimension to the submicron range significantly increases Cu-based interconnect and contact resistance. To address this challenge, topological semimetals, such as CoSi and CoPt, have been investigated as potential replacements for chip-level interconnects due to their surface-dominated transport properties [64,65,66]. Additionally, system-level NbAs interconnects have been explored [64]; however, the application of topological semimetals for chip-to-wafer (C2W) and wafer-to-wafer (W2W) bonding—where material interactions play a crucial role—remains largely unexplored. Given their superior electron mobility at the nanoscale compared to copper, topological semimetals hold promise for C2W and W2W applications as contact sizes continue to shrink to deep submicron or nanoscale dimensions. Looking further ahead, signal transmission at extremely small interconnect scales may shift toward spin wave propagation [67], potentially rendering conventional Cu-based C2W and W2W interconnects obsolete.

## Figures and Tables

**Table 1:** Summary of the bonding methods and key features discussed.

Bonding Method	Pitch (μm)	Bonding Conditions	Key Features	Reference
Direct hybrid bonding (Surface plasma activation)	5	Room temp., 0.3 MPa	Defect-free and good grinding resistant	[32]
Cluster-Ag passivation	~15	150 °C	3.77 MPa (Pull test) and 21.95 MPa (Shear test)	[37]
N2 plasma treatment/Passivation	0.4–0.7	NA	Void-free, well Cu-Cu inter-diffused and well-aligned	[39]
Pillar–Concave Structure	~25	200 °C, 500 MPa	Early breakdown of polyimide/concave interface in shear test	[41]
All-Cu 3D interconnect	2	<200 °C	Simple process	[47]
High throughput two-stage TCB	7	300 °C and 100 MPa or 400 °C and 50 MPa (annealing)	No bonding interface observed	[43]
Cu-Cu Wiring	1.4	NA	Simple process	[46]
Unequal submicron-sized pads	0.7	230 °C to 350 °C (post-bond annealing)	Complicated in design, variation in contact resistance	[48]
Crystalline (111)-Oriented Nano-twinned Cu	20	200 °C, 1.06 MPa	Thermal and electrical stability up to 375 °C	[54]
Crystalline metastable microstructure	--	200 °C	To monitor grain growth on the bonding surface	[55,56]
Photosensitive polyimide adhesive	20	250–350 °C, 2–6 MPa	Shear test 4–4.5 MPa	[61]
Citric or ascorbic aqueous treatment	20	250 °C, 5 MPa	Shear test > 20 MPa	[62]
Fly cutting (Cu/polymer hybrid bonding)	--	200°C	No voids at PI-PI interface, some seams at Cu-Cu interface	[63]

## Data Availability

No new data were created or analyzed in this study.
